# Calsequestrin 1 Is an Active Partner of Stromal Interaction Molecule 2 in Skeletal Muscle

**DOI:** 10.3390/cells10112821

**Published:** 2021-10-20

**Authors:** Seung Yeon Jeong, Mi Ri Oh, Jun Hee Choi, Jin Seok Woo, Eun Hui Lee

**Affiliations:** 1 Department of Physiology, College of Medicine, The Catholic University of Korea, Seoul 06591, Korea; tjdus0560@catholic.ac.kr (S.Y.J.); mroh@catholic.ac.kr (M.R.O.); junhee@catholic.ac.kr (J.H.C.); 2 Department of Biomedicine & Health Sciences, Graduate School, The Catholic University of Korea, Seoul 06591, Korea; 3 Department of Physiology, David Geffen School of Medicine, UCLA, Los Angeles, CA 10833, USA; ulbojs@gmail.com

**Keywords:** CASQ1, STIM2, SOCE, skeletal muscle

## Abstract

Calsequestrin 1 (CASQ1) in skeletal muscle buffers and senses Ca^2+^ in the sarcoplasmic reticulum (SR). CASQ1 also regulates store-operated Ca^2+^ entry (SOCE) by binding to stromal interaction molecule 1 (STIM1). Abnormal SOCE and/or abnormal expression or mutations in CASQ1, STIM1, or STIM2 are associated with human skeletal, cardiac, or smooth muscle diseases. However, the functional relevance of CASQ1 along with STIM2 has not been studied in any tissue, including skeletal muscle. First, in the present study, it was found by biochemical approaches that CASQ1 is bound to STIM2 via its 92 N-terminal amino acids (C1 region). Next, to examine the functional relevance of the CASQ1-STIM2 interaction in skeletal muscle, the full-length wild-type CASQ1 or the C1 region was expressed in mouse primary skeletal myotubes, and the myotubes were examined using single-myotube Ca^2+^ imaging experiments and transmission electron microscopy observations. The CASQ1-STIM2 interaction via the C1 region decreased SOCE, increased intracellular Ca^2+^ release for skeletal muscle contraction, and changed intracellular Ca^2+^ distributions (high Ca^2+^ in the SR and low Ca^2+^ in the cytosol were observed). Furthermore, the C1 region itself (which lacks Ca^2+^-buffering ability but has STIM2-binding ability) decreased the expression of Ca^2+^-related proteins (canonical-type transient receptor potential cation channel type 6 and calmodulin 1) and induced mitochondrial shape abnormalities. Therefore, in skeletal muscle, CASQ1 plays active roles in Ca^2+^ movement and distribution by interacting with STIM2 as well as Ca^2+^ sensing and buffering.

## 1. Introduction

Skeletal muscle contracts or relaxes to move the body or to sustain body postures, which is dependent on the cytosolic Ca^2+^ level of myotubes (i.e., skeletal muscle cells) [1,2,3,4,5]. A transient elevation of cytosolic Ca^2+^ levels couples action potentials on the t-tubule membrane with skeletal muscle contractions (called excitation-contraction (EC) coupling). During skeletal EC coupling, dihydropyridine receptor (DHPR, a voltage-gated Ca^2+^ channel on the t-tubule membrane) is activated by sensing action potentials in response to acetylcholine and changes its conformation. Active DHPR activates ryanodine receptor type 1 (RyR1, an internal Ca^2+^ channel on the sarcoplasmic reticulum (SR) membrane) via physical interactions. Active RyR1 releases Ca^2+^ from the SR (an internal Ca^2+^ store) to the cytosol, and Ca^2+^ turns on contractile proteins to evoke skeletal muscle contractions. In addition to the major role of the SR of storing internal Ca^2+^, mitochondria also have a role in storing internal Ca^2+^ in skeletal muscle [6,7]. To relax skeletal muscle, cytosolic Ca^2+^ moves back to the SR via sarcoplasmic/endoplasmic reticulum Ca^2+^-ATPase1a (SERCA1a, a Ca^2+^ pump in the SR membrane) [8]. The coordinated arrangement of the proteins mentioned above in the triad junction (which is composed of two t-tubule membranes and an SR between them, such as a sandwich) is required for the transient elevation or removal of cytosolic Ca^2+^ during the contraction or relaxation of skeletal muscle [9,10,11].

Cytosolic Ca^2+^ that is used for skeletal muscle contraction is supplied from the extracellular space as well as from the SR [1,2,12,13]. Store-operated Ca^2+^ entry (SOCE) is a method for extracellular Ca^2+^ to enter into skeletal myotubes. Stromal interaction molecule 1 (STIM1, a Ca^2+^ sensor on the SR membrane) and Orai1 (an extracellular Ca^2+^ entry channel on the t-tubule membrane) are the main SOCE-mediating proteins. In short, during skeletal SOCE, STIM1 senses the depletion of Ca^2+^ from the SR and then interacts with Orai1 to form functional puncta. Punctum formation activates Orai1 to allow extracellular Ca^2+^ to enter into the cytosol. In addition to the main role of Orai1 and STIM1 in SOCE, other proteins also participate in skeletal SOCE [1,2,14]. Canonical-type transient receptor potential cation channels (TRPCs) mediate Ca^2+^ entry via the SOCE mechanism [15,16,17,18]. STIM2 is a homolog of STIM1 and plays redundant roles, to some degree, in SOCE and the terminal differentiation of skeletal muscle [19,20,21,22]. However, few studies have investigated the roles of STIM2 in skeletal muscle.

CASQ1, the major isoform in adult fast-twitch skeletal muscles, is enriched in the SR [23]. CASQ1 buffers Ca^2+^ in the SR with a low-affinity and high-capacity Ca^2+^-binding ability (40~50 moles or maximum ~80 moles of Ca^2+^/1 mole of CASQ1) [23,24]. This ability is important for both the preparation of rapidly releasable Ca^2+^ from the SR during skeletal muscle contraction and the efficient uptake of Ca^2+^ to the SR during skeletal muscle relaxation, which occurs without harmful osmotic effects due to the storage of high [Ca^2+^] in the SR. In addition to the Ca^2+^-buffering ability, CASQ1 has a Ca^2+^-sensing ability. CASQ1 senses the degree of Ca^2+^ depletion from the SR and modulates Ca^2+^ release from the SR to the cytosol via RyR1 in a conformation (i.e., polymerization)-dependent manner [25,26].

Dysregulation of SOCE and/or mutations of CASQ1 have been reported in human patients or animal models with skeletal muscle diseases such as tubular aggregate myopathy (TAM) or malignant hyperthermia [23,27,28,29,30]. The involvement of STIM1 and/or STIM2 in various human diseases, including skeletal muscle diseases, has also been reported [1,2,31,32,33]. It was reported that the C-terminus of CASQ1 binds to STIM1 and inhibits SOCE in heterologous expression systems or C2C12 myotubes [27,34,35]. However, the existence of an interaction between CASQ1 and STIM2 and the functional relevance of CASQ1 in conjunction with STIM2 in skeletal muscle remains unknown.

Therefore, the present study is focused on verifying whether CASQ1 binds to STIM2 using biochemical approaches, identifying STIM2-binding region on CASQ1 if CASQ1 binds to STIM2, and examining the functional relevance of the CASQ1-STIM2 interaction in skeletal muscle using mouse primary skeletal myotubes (instead of a heterologous expression system involving variations in expression and artifacts), single-myotube Ca^2+^ imaging experiments, and transmission electron microscopy (TEM) observations.

## 2. Materials and Methods

### 2.1. Ethical Approval

The methods were carried out in accordance with the regulations and guidelines of the College of Medicine at The Catholic University of Korea. The site where the animal work took place and all surgical interventions, including pre- and post-surgical animal care, were carried out in accordance with the Laboratory Animals Welfare Act, the Guide for Care and Use of Laboratory Animals, and the Guidelines and Policies for Rodent Survival Surgery approved by the Institutional Animal Care and Use Committee of the College of Medicine at The Catholic University of Korea (The ethic approcal code is 2017-0117-01). All protocols for the experiments were approved by the Committee of the College of Medicine at The Catholic University of Korea.

### 2.2. cDNA Construction and Expression of the GST-CASQ1 or GST-CASQ1 Regions

cDNA of mouse CASQ1 was obtained from OriGene Technologies, Inc. (Rockville, MD, USA, #MR206274). To prepare cDNA for GST-tagged full-length CASQ1 (GST-CASQ1) or CASQ1 regions, oligonucleotide primers were designed based on mouse CASQ1 (GenBank accession number: NM_009813) (Supplementary Tables S1 and S2). With the primers, PCR was performed (30 cycles at 95 °C for 45 s, 63 °C for 45 s, and 68 °C for 90 s). The PCR products were subcloned into the pGEX-4T-1 vector. GST-CASQ1 or GST-CASQ1 regions were expressed in *E. coli* (DH5α) using 0.1 mM isopropyl-β-D-thiogalactopyranoside (Sigma–Aldrich, St. Louis, MO, USA), as previously described [22,36,37]. For expressing the full-length wild-type CASQ1 (WT CASQ1) in mouse primary skeletal myotubes, full-length CASQ1 in the pGEX-4T-1 vector was subcloned into the pCMS-RFP vector using EcoR I and Not I enzyme sites. For the C1 region, the PCR products using primers in Supplementary Table S3 were subcloned into the pCMS-RFP vector.

### 2.3. Triad Sample Preparation and the Binding Assay of CASQ1 Regions with Triad Proteins

Triad vesicles (that are enriched with triad proteins mediating intra- and extracellular Ca^2^^+^ movements in skeletal muscle, including STIM2 [1,2,3,5]) were prepared and solubilized to create triad samples, as previously described [38,39,40,41]. Binding assays were performed as previously described [37]. Briefly, affinity beads were prepared by immobilizing the GST-CASQ1 or GST-CASQ1 regions on GST beads (Amersham, GE Healthcare Biosciences, Pittsburgh, PA, USA). The affinity beads were then incubated with 150 μg of the triad sample for 6 h at 4 °C. The proteins that were bound to the affinity beads were separated on a 10% SDS–PAGE gel and subjected to an immunoblot assay.

### 2.4. Cell Culture and Expression of the WT CASQ1 or C1 Region

Mouse primary skeletal myoblasts that were derived from mouse skeletal muscle using a single-cell cloning method were expanded and differentiated into myotubes, as previously described [41,42,43,44,45]. For differentiating the skeletal myoblasts to myotubes, myoblasts were replated on different plates coated with Matrigel (BD Biosciences, Sparks Glencoe, MD, USA, 6-well plates for the TEM observation or immunocytochemistry experiment, 96-well plates for the single-myotube Ca^2^^+^ imaging experiment, or 10-cm plates for other experiments). After three days of culture under differentiation conditions, premature myotubes were transfected with an empty vector as a control or cDNA encoding the WT CASQ1 or C1 region (a mixture of 30 µL of FuGENE6 (Promega, Madison, WI, USA) and 20 μg of cDNA per 10-cm dish, or the same ratio of components in the well of other plates, for 3 h). Mature myotubes were either observed, imaged, or disrupted at 36 h post-transfection for further experiments, at which time approximately 60% of the myotubes had been transfected, as estimated by the RFP signal. All reagents that were used for the cell cultures were obtained from Invitrogen (Waltham, MA, USA).

### 2.5. Coimmunoprecipitation and Immunoblot Assays

Mouse primary skeletal myotubes were solubilized in lysis buffer, as previously described [10,22,38,41,42,44]. For the coimmunoprecipitation assay [22,41,42,44], solubilized myotube lysate (100 µg of total protein) and anti-CASQ1 (Affinity BioReagents, Golden, CO, USA) or anit-Orai1 antibody (Abcam, Cambridge, MA, USA) were used. The immunoprecipitate was subjected to immunoblot assays with anti-CASQ1, anti-Orai1, or anti-STIM2 antibody (Abcam). For the immunoblot assay, solubilized myotube lysate (10 μg of total protein) was subjected to SDS–PAGE (8, 10, or 12% gel) [10,22,38,41,42,44,46]. The anti-RyR1, anti-SERCA1a, anti-CASQ1, anti-CaM1, anti-JP1, and anti-JP2 antibodies were obtained from Affinity BioReagents. The anti-TRPC1, anti-TRPC3, anti-TRPC4, and anti-TRPC6 antibodies were obtained from Alomone Laboratories (Jerusalem, Israel). The anti-TRIM32, anti-MyoD, and anti-myogenin antibodies were obtained from Santa Cruz Biotechnology (Dallas, TX, USA). The anti-DHPR, anti-STIM1, anti-STIM2, and anti-α-actin antibodies were obtained from Abcam.

### 2.6. Immunocytochemistry and Width Measurement

For the immunocytochemistry experiments, myotubes were fixed in cold methanol (−20 °C) for 30 min, permeabilized with 0.05% Tween 20 phosphate-buffered saline for 1 min, and stained with anti-GFP (for detecting RFP-tagged proteins) and Cy3-conjugated secondary antibodies, as previously described [10,38,41,42,44]. Myotube widths (one criterion that is used to evaluate the degree of skeletal myotube formation) were measured using the ImageJ program, as previously described [10,38,41,42,45,46].

### 2.7. Single-Myotube Ca^2^^+^ Imaging

Single-myotube Ca^2^^+^ imaging was performed using a high-speed monochromator with a 75 W xenon lamp (FSM150Xe, Bentham Instruments, Reading, Berkshire, UK) and an inverted-stage microscope (Nikon Eclipse TS100, Nikon Instruments, Inc., Melville, NY, USA). Mouse primary skeletal myotubes were loaded with 5 μM fura-2-AM (Invitrogen) for the measurement of cytosolic [Ca^2^^+^] or fluo-4-AM (Invitrogen) for other measurements in imaging buffer (25 mM HEPES, pH 7.4, 125 mM NaCl, 5 mM KCl, 2 mM KH_2_PO_4_, 2 mM CaCl_2_, 6 mM glucose, 1.2 mM MgSO_4_, and 0.05% BSA) at 37 °C for 45 min, as previously described [10,22,38,41,42,44,45,46]. Either caffeine (20 mM) or KCl (60 mM) was dissolved in the imaging buffer and applied to myotubes via an autoperfusion system (AutoMate Scientific, St. Berkeley, CA, USA). The data were analyzed or displayed using image acquisition and analysis software (High-Speed InCyt Im2 for cytosolic [Ca^2^^+^] and Im1 for other results, v5.29, Intracellular Imaging Inc., Cincinnati, OH, USA). To measure releasable Ca^2^^+^ from the SR, thapsigargin (TG, 2.5 μM) dissolved in dimethyl sulfoxide (DMSO, <0.05%, no effect by itself) in the absence of extracellular Ca^2^^+^ was applied to myotubes. For the SOCE measurement, Ca^2^^+^ in the SR was depleted with TG (2.5 μM) in the absence of extracellular Ca^2^^+^, and once the cytosolic Ca^2^^+^ level returned to baseline, Ca^2^^+^ (2 mM) was added to myotubes to measure SOCE. To analyze Ca^2^^+^ movement, the peak amplitudes (which exhibited similar changes in peak areas) were measured. For long-term Ca^2^^+^ movements such as SOCE or the response to TG, the area under the curve was analyzed. To analyze the initial rate of responses, the slope at the rising phase of the response was examined by a linear equation that was obtained from a linear fitting of the rising phase [10,22,41,44,45]. Reagents that were used for the single-myotube Ca^2^^+^ imaging were obtained from Sigma–Aldrich.

### 2.8. TEM Observation

For TEM observations, myotubes were fixed, embedded in epoxy resin (Epon 812), sectioned using an ultramicrotome (70–80 nm, Ultracut UCT ultramicrotome, Leica, Buffalo Grove, IL, USA), and examined under TEM (JEM1010, JEOL Ltd., Peabody, MA, USA) at 60 kV, as previously described [10,41]. To analyze the length of mitochondria, the mitochondrial length in a unit area (37.5 μm × 25.0 μm) was measured using ImageJ software (Version 1.53m, NIH, Bethesda, MD, USA).

### 2.9. Presentation of the Three-Dimensional (3D) Structure of CASQ1

The 3D structure of human CASQ1 (PDB ID: 3UOM) is presented as a ribbon diagram using iCn3D (NCBI’s web-based 3D structure viewer) [47].

### 2.10. Statistical Analysis

**Figure 1:**
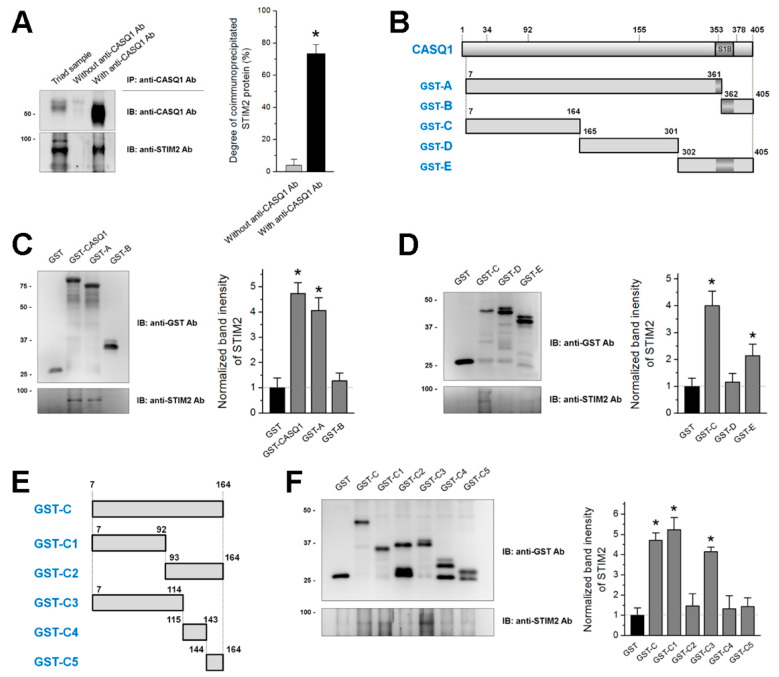
Coimmunoprecipitation of CASQ1 with STIM2 and the binding assay of CASQ1 regions to STIM2: (**A**) Triad samples obtained from rabbit skeletal muscle were subjected to a coimmunoprecipitation assay with anti-CASQ1 antibody. ‘Triad sample’ indicates a simple immunoblot of the triad sample. ‘Without anti-CASQ1 Ab’ indicates a reaction without an anti-CASQ1 antibody. Three independent experiments were conducted, and a representative result is presented. The degree of coimmunoprecipitated STIM2 to total STIM2 is presented as histograms. STIM2 was successfully coimmunoprecipitated with CASQ1. IB, IP, or Ab indicates immunoblot, immunoprecipitation, or antibody, respectively. * Significant difference was compared with ‘without anti-CASQ1 Ab’ (*p* < 0.05). (**B**,**E**) Schematic diagrams of mouse CASQ1 regions are presented. Numbers indicate the sequence of amino acids. The S1B region is presented according to a previous report [35]. (**C**,**D**,**F**) The bound proteins that were obtained from the binding assays of GST-CASQ1 or GST-CASQ1 regions with triad samples were subjected to immunoblot assays with anti-GST (to detect GST-CASQ1 or GST-CASQ1 regions) or anti-STIM2 antibody. GST was used as a negative control. Three independent experiments were conducted. The relative amount of STIM2 to the corresponding amount of the CASQ1 region is presented as histograms on the right-hand side. The value for the relative amount of STIM2 to GST was regarded as 1, and others were normalized by this value. * Significant difference compared with GST (*p* < 0.05).

**Figure 2:**
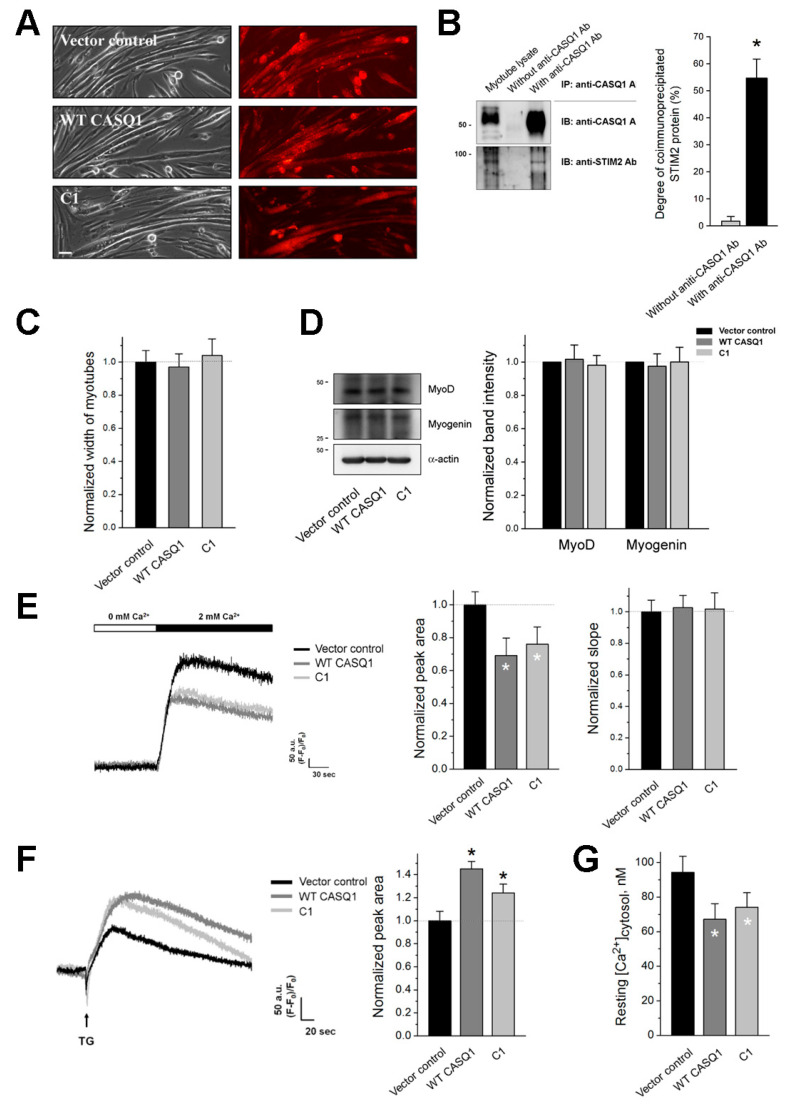
SOCE, Ca^2+^ amount in the SR, and cytosolic Ca^2+^ level in WT CASQ1 or C1-expressing mouse skeletal myotubes: (**A**) Mouse primary skeletal myotubes that were transfected with cDNA of empty vector (vector control), full-length wild-type CASQ1 (WT CASQ1), or C1 region (C1) were subjected to immunocytochemistry using anti-GFP antibodies. The bar represents 100 µm. (**B**) The lysate of WT CASQ1 or C1-expressing myotubes was subjected to a coimmunoprecipitation assay with anti-CASQ1 antibody, and the immunoprecipitate was subjected to immunoblot analysis with anti-CASQ1 or anti-STIM2 antibody. ‘Myotube lysate’ indicates a simple immunoblot of the myotube lysate. ‘Without anti-CASQ1 Ab’ indicates a reaction without an anti-CASQ1 antibody. Three independent experiments were conducted, and a representative result is presented. The degree of coimmunoprecipitated STIM2 to total STIM2 is presented as histograms. STIM2 was successfully coimmunoprecipitated with CASQ1. IB, IP, or Ab indicates immunoblot, immunoprecipitation, or antibody, respectively. * Significant difference was compared with ‘without anti-CASQ1 Ab’ (*p* < 0.05). (**C**) Myotube widths were measured. The normalized mean values of each to the mean value of the vector control are summarized as histograms. There was no significant difference in the width of the myotubes. (**D**) The myotube lysate was subjected to immunoblot assays with antibodies against MyoD or myogenin. *α*-actin was used as a loading control. Three independent experiments per protein were conducted. The expression level of each protein normalized to the mean value of the vector control is presented as histograms (mean ± SEM for three independent experiments, Supplementary Table S4). There was no significant difference in their expression level. (**E**) SOCE was measured in the myotubes by depleting the SR with TG in the absence of extracellular Ca^2+^ and applying extracellular Ca^2+^ to the myotubes. The results are summarized as histograms for the area under the peak (left-hand side) or the slope in the rising phase of SOCE (right-hand side). There was no significant difference in the slopes of the myotubes. (**F**) Ca^2+^ amount in the SR was measured in the myotubes by treatment with TG in the absence of extracellular Ca^2+^. The results are summarized as histograms for the area under the peak. A representative trace for each group is shown (E and F). The experimental mean values were normalized to the mean values of the vector control (**E**,**F**). (**G**) Cytosolic Ca^2+^ levels at rest were measured in the myotubes, and the mean values are summarized as histograms. The values are presented as the mean ± SEM for the number of myotubes shown in parentheses in Table 1. * Significant difference compared with vector control (*p* < 0.05).

The results are presented as the mean ± SEM for the number of myotubes or experiments shown in the figure legends or the table parentheses. Significant differences were analyzed using an unpaired *t*-test (for Figure 1A and Figure 2B) or one-way ANOVA (for others) (GraphPad InStat, v2.04, GraphPad Software, San Diego, CA, USA). The differences were considered to be significant for *p* < 0.05. The graphs were prepared using Origin 2019b (OriginLab, Northampton, MA, USA).

## 3. Results

### 3.1. N-Terminal Region of CASQ1 Binds to STIM2

To examine whether CASQ1 binds to STIM2, a triad sample obtained from rabbit skeletal muscle was subjected to a coimmunoprecipitation assay with anti-CASQ1 antibody, and the immunoprecipitate was subjected to immunoblot analysis with anti-CASQ1 or anti-STIM2 antibody (Figure 1A). STIM2 coimmunoprecipitated with CASQ1, suggesting that CASQ1 binds to STIM2.

To identify which region of CASQ1 is involved in binding to STIM2, cDNAs for five regions of mouse CASQ1 (A to E in Figure 1B) were constructed as a GST-tagged form and expressed in E. coli. The STIM1-binding (S1B) region in mouse CASQ1 is indicated in Figure 1B (353–378 amino acids that correspond to the S1B region of human CASQ1 [35]). To examine whether the S1B region of CASQ1 also participates in binding to STIM2, the S1B region was disrupted by dividing CASQ1 into two regions (GST-A and GST-B) or it was left intact (GST-E). First, GST-A or GST-B was subjected to a binding assay with STIM2 (Figure 1C). GST-CASQ1 (i.e., full-length CASQ1) successfully bound to STIM2 and was used as a positive control. GST alone was used as a negative control. GST-A, but not GST-B, bound to STIM2. Second, GST-C, GST-D, or GST-E was subjected to a binding assay with STIM2 (Figure 1D). GST-C bound to STIM2, but GST-D did not bind to STIM2. GST-E with intact S1B was also significantly bound to STIM2, but much less than GST-C. These results suggest that the C region of CASQ1 mainly contributes to the binding of CASQ1 to STIM2.

To narrow the binding region of CASQ1 to STIM2 within the C region, smaller regions of the C region were constructed (Figure 1E) and subjected to a binding assay with STIM2 (Figure 1F). GST-C (full-length C region) was used as a positive control. GST-C1 or GST-C3 bound to STIM2 (C3 region covers the C1 region), but others were not. These results suggest that the C1 region (92 N-terminal amino acids of CASQ1) plays a major role in the binding of CASQ1 to STIM2.

### 3.2. The CASQ1 or C1 Region Decreases SOCE and Changes Intracellular Ca^2+^ Distribution

To examine the functional roles of the CASQ1-STIM2 interaction in skeletal muscle, full-length wild-type CASQ1 (WT CASQ1) or the C1 region (C1) was expressed in mouse primary skeletal myotubes instead of heterologous expression systems to avoid possible artifacts and variations in expression introduced by the cell system. Myotubes that were transfected with empty vectors were used as a negative control (also for subsequent experiments). WT CASQ1 or C1 was successfully expressed (Figure 2A). To confirm whether CASQ1 also binds to STIM2 in mouse skeletal muscle as it does in rabbit skeletal muscle, the lysate of WT CASQ1 or C1-expressing myotubes was subjected to a coimmunoprecipitation assay with anti-CASQ1 antibody, and the immunoprecipitate was subjected to immunoblot analysis with anti-CASQ1 or anti-STIM2 antibody (Figure 2B). STIM2 coimmunoprecipitated with CASQ1, suggesting that CASQ1 also binds to STIM2 in mouse skeletal muscle. The width of the myotubes (which allows estimation of the degree of terminal differentiation) was measured, and there was no significant difference in the width by the expression of WT CASQ1 or C1 (Figure 2C and Table 1). In addition, expression levels of two myogenic markers, MyoD, and myogenin were examined by immunoblot assays, and there was no significant change in their expression levels by the expression of WT CASQ1 or C1 (Figure 2D and Supplementary Table S4). These results suggest that the terminal differentiation of myotubes was not significantly affected by the CASQ1-STIM2 interaction. The myotubes that expressed WT CASQ1 or C1 were subjected to further experiments.

To examine SOCE, Ca^2+^ in the SR of the myotubes in the absence of extracellular Ca^2+^ was depleted with TG to avoid extracellular Ca^2+^ entry during SR depletion, and extracellular Ca^2+^ was then applied to the myotubes to measure SOCE using single-myotube Ca^2^^+^ imaging (Figure 2E and Table 1). Both WT CASQ1 and C1 significantly decreased SOCE without a change in the slope at the rising phase of SOCE (i.e., without a change in the initial rate of SOCE). This result suggests that the CASQ1-STIM2 interaction decreased SOCE in skeletal muscle.

To address the aftereffects of the decrease in SOCE, Ca^2+^ levels in the myotubes were measured. First, the releasable Ca^2+^ from the SR (which allows the amount of Ca^2+^ in the SR to be estimated) in the absence of extracellular Ca^2+^ was measured by depleting the SR with TG (Figure 2F and Table 1). The absence of extracellular Ca^2+^ allows an assessment of Ca^2+^ releasable exclusively from the SR without extracellular Ca^2+^ entry during SR depletion. Interestingly, the Ca^2+^ amount in the SR of the myotubes was significantly increased by both WT CASQ1 and C1 compared with that by the vector control. Second, cytosolic Ca^2+^ levels at rest were measured in the myotubes (Figure 2G and Table 1). Cytosolic Ca^2+^ levels were significantly decreased by both WT CASQ1 and C1 compared with those by the vector control. These results suggest that the intracellular Ca^2+^ distribution between the SR and the cytosol was changed by the CASQ1-STIM2 interaction, in addition to the decrease in SOCE.

### 3.3. The CASQ1 or C1 Region Increases Intracellular Ca^2+^ Release through RyR1 for Muscle Contraction

**Figure 3:**
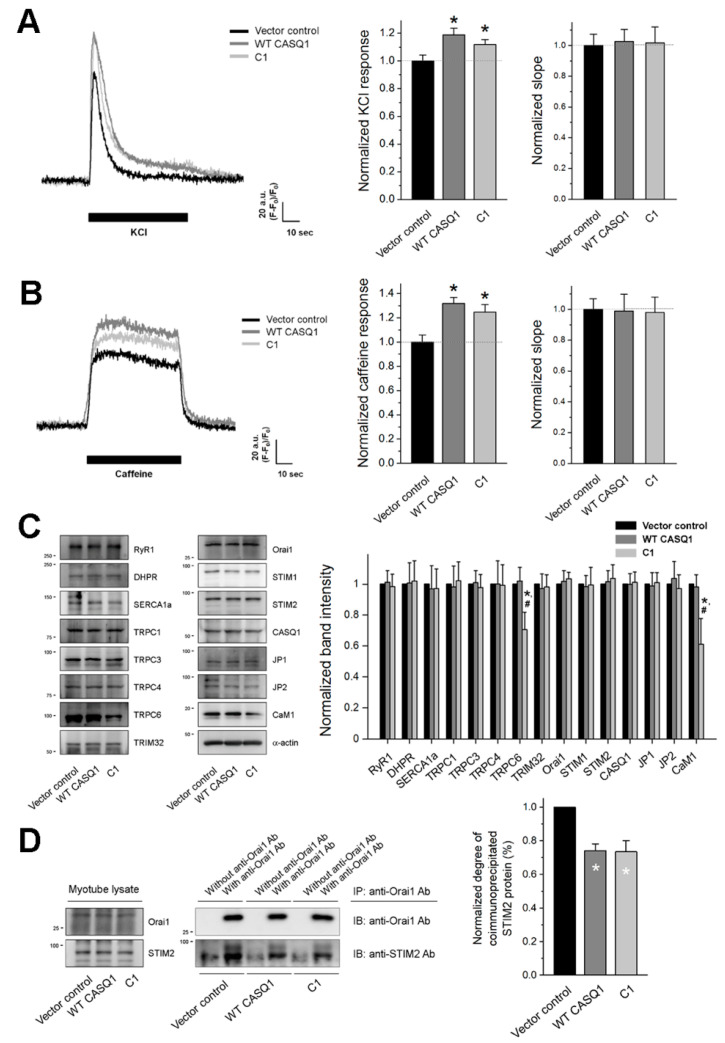
Intracellular Ca^2+^ release for skeletal muscle contraction, expression levels of various proteins, and coimmunoprecipitation of Orai1 with STIM2: KCl (**A**) or caffeine (**B**) was applied to the myotubes, and intracellular Ca^2+^ release from the SR to the cytosol through RyR1 was measured. A representative trace for each group is shown. Histograms show the normalized peak amplitude (left-hand side) or the slope in the rising phase of the peak to the mean value of the vector control (right-hand side). There was no significant difference in the slopes of the myotubes. The results are presented as the mean ± SEM for the number of myotubes shown in parentheses in Table 1. (**C**) The myotube lysate was subjected to immunoblot assays with antibodies against fifteen proteins. *α*-actin was used as a loading control. Three independent experiments per protein were conducted. CASQ1, endogenous CASQ1; JP, junctophilin. The expression level of each protein normalized to the mean value of the vector control is presented as histograms (mean ± SEM for three independent experiments, Supplementary Table S4). * Significant difference compared with vector control (*p* < 0.05). ^#^ Significant difference compared with WT CASQ1 (*p* < 0.05). (**D**) The myotube lysate was subjected to a coimmunoprecipitation assay with anti-Orai1 antibody, and the immunoprecipitate was subjected to immunoblot analysis with anti-Orai1 or anti-STIM2 antibody. ‘Myotube lysate’ on the left-hand side indicates a simple immunoblot of the myotube lysate. ‘Without anti-Orai1 Ab’ indicates a reaction without an anti-Orai1 antibody. Three independent experiments were conducted, and a representative result is presented. The degree of coimmunoprecipitated STIM2 (i.e., the degree of coimmunoprecipitated STIM2 to total STIM2) normalized to the mean value of the vector control is presented as histograms. IB, IP, or Ab indicates immunoblot, immunoprecipitation, or antibody, respectively. * Significant difference was compared with vector control (*p* < 0.05).

To examine intracellular Ca^2+^ release for skeletal muscle contraction, a membrane depolarizer (KCl that induces the depolarization of the membrane, such as that during EC coupling) was applied to the myotubes, and intracellular Ca^2^^+^ release from the SR to the cytosol through RyR1 was measured. Intracellular Ca^2^^+^ release in response to membrane depolarization in skeletal myotubes mimics intracellular Ca^2^^+^ release that is needed for the contraction of skeletal muscle [10,41,42]. Both WT CASQ1 and C1 significantly increased Ca^2^^+^ release in response to membrane depolarization without a change in the initial rate of the Ca^2^^+^ release (no change in the slope at the rising phase of Ca^2^^+^ release compared with that of the vector control, Figure 3A and Table 1). To address the reason for the increases in Ca^2^^+^ release, the activity of RyR1 was assessed by applying a direct agonist of RyR1 (caffeine [48]) to the myotubes (Figure 3B and Table 1). Ca^2^^+^ release in response to caffeine showed a similar pattern as membrane depolarization. These results suggest that the increases in Ca^2^^+^ release in response to membrane depolarization are not due to a change in DHPR activity or the coupling between DHPR and RyR1 but due to the increased RyR1 activity by the CASQ1-STIM2 interaction.

To assess the expression levels of fifteen proteins that regulate or mediate SOCE or EC coupling in skeletal muscle, immunoblot assays were conducted with the myotube lysate (Figure 3C and Supplementary Table S4). There was no significant change in the expression level of the key EC coupling-mediating proteins (DHPR, RyR1, SERCA1a, or endogenous CASQ1) or structural proteins for the formation of triad junctions (JP1 or JP2). There was also no significant change in SOCE-mediating proteins (Orai1, STIM1, or STIM2). In addition, there was no significant change in tripartite motif-containing protein 32 (TRIM32, which controls myogenic differentiation by regulating c-Myc [49]), which again confirms that the terminal differentiation of myotubes was not significantly affected by the CASQ1-STIM2 interaction. These results confirm that neither the increase nor decrease in the Ca^2^^+^ movements or distribution of the myotubes that expressed WT CASQ1 or C1 was caused by a change in the expression level of proteins that regulate or mediate SOCE or EC coupling but were instead due to the CASQ1-STIM2 interaction.

To examine whether the expression of WT CASQ1 or C1 (i.e., CASQ1-STIM2 or C1-STIM2 interaction) reduces Orai1-STIM2 interaction, the myotube lysate was subjected to a coimmunoprecipitation assay with anti-Orai1 antibody, and the immunoprecipitate was subjected to immunoblot analysis with anti-Orai1 or anti-STIM2 antibody (Figure 3D). Significantly, less amount of STIM2 was coimmunoprecipitated with Orai1 in WT CASQ1 or C1-expressing myotubes compared with vector control.

### 3.4. C1 Region Induces Mitochondrial Shape Abnormalities

**Figure 4:**
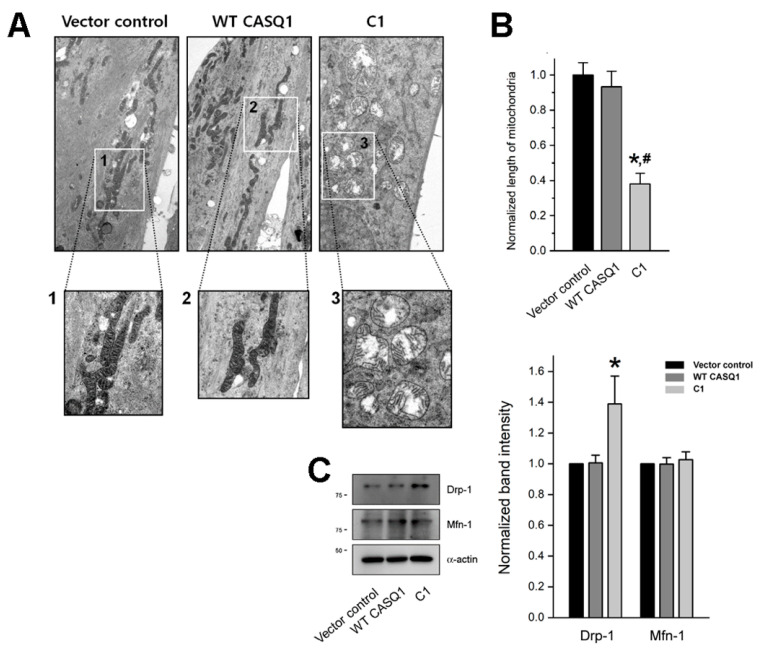
Abnormal mitochondria in the shape and the expression level of Drp-1 or Mfn-1: (**A**) Mitochondria in the myotubes were observed using TEM. Areas in the numbered boxes (from 1 to 3) were enlarged. Swelling mitochondria were found in C1-expressing myotubes. The bar represents 2 μm. (**B**) The length of the swelling mitochondria was measured, and the results are summarized as histograms. The values were normalized to the mean values of the vector control. The values are presented as the mean ± SEM for the number of mitochondria in Table 2. (**C**) The myotube lysate was subjected to immunoblot assays with antibodies against Drp-1 or Mfn-1. *α*-actin was used as a loading control. Three independent experiments per protein were conducted. The expression level of each protein normalized to the mean value of the vector control is presented as histograms (Table 2). * Significant difference compared with vector control (*p* < 0.05). ^#^ Significant difference compared with WT CASQ1 (*p* < 0.05).

Interestingly, among four TRPCs that are known to be expressed in skeletal muscle (TRPC1, TRPC3, TRPC4, and TRPC6 [18]), the expression of TRPC6 was significantly decreased by the C1 region compared with that by vector control or WT CASQ1, and the expression of calmodulin 1 (CaM1) was also decreased (Figure 3C and Supplementary Table S4). To assess the effect or cause of the decrease in TRPC6 or CaM1 expression, myotubes were observed by TEM (Figure 4A). Swelling mitochondria were found in C1-expressing myotubes. The cristae in the swelling mitochondria were disrupted (enlarged image 3 in Figure 4A). The swelling mitochondria were significantly short (Figure 4B). These results suggest that the C1 region severely affected mitochondrial shapes.

To examine the cause of the short and swelling mitochondria, the expression level of proteins that mediate mitochondrial fission or fusion was examined by immunoblot assays using the lysate of C1-expressing myotubes. The expression of the dynamin-1-like protein (Drp-1), which mediates the fission process of mitochondria, was significantly increased (Figure 4C, Table 2). However, the expression of fusion-mediating mitofusin-1 (Mfn-1) was not changed. These results suggest that excessive fission of mitochondria could be one of the reasons for the short and swelling mitochondria in C1-expressing myotubes.

**Figure 5:**
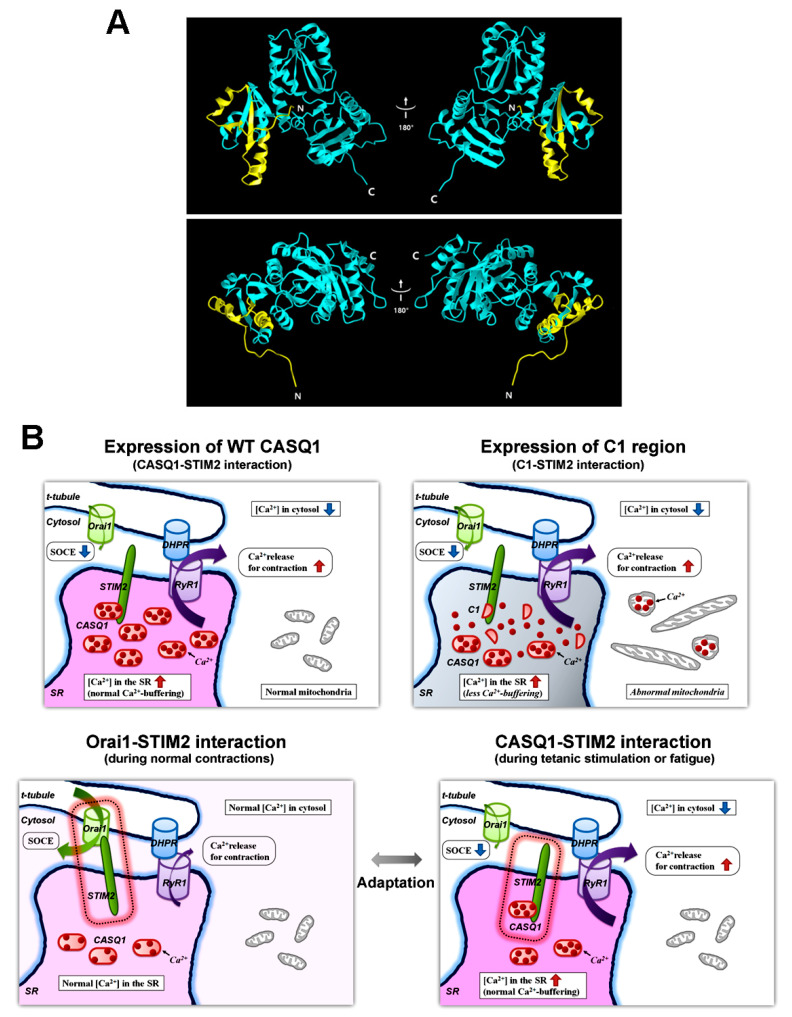
3D structure of human CASQ1 and an adaptation model to situations with repetitive and/or long-term contractions of skeletal muscle: (**A**) 3D structure of human CASQ1 (PDB ID: 3UOM, starting from 3 to 387 amino acids) is presented as a ribbon diagram. The C1 region of CASQ1, which is involved in binding to STIM2, is colored yellow. N or C indicates the N- or C-terminus, respectively. The right images are the 180° rotated images of the left images along the vertical axis. Images in the lower panel are the top view of the images in the upper panel. (**B**) The overall results of this study are summarized as schematic diagrams in the upper panel. In the lower panel, ‘an adaptation model to situations with repetitive and/or long-term contractions of skeletal muscle’, such as tetanic stimulation or fatigue, is presented.

The N-terminal C1 region of CASQ1 is colored yellow in the 3D structure of human CASQ1 (Figure 5A). The C1 region is located on a freely accessible surface of CASQ1, which is favorable for the interaction of CASQ1 with STIM2. The overall results of this study are summarized in the upper panel of Figure 5B.

## 4. Discussion

In the present study, we found that the N-terminal region of CASQ1 (C1 region) binds to STIM2. Expression of the C1 region in mouse primary skeletal myotubes induces similar influences on the expression of full-length CASQ1, i.e., the CASQ1-STIM2 interaction increases intracellular Ca^2+^ release through RyR1 for skeletal muscle contraction and Ca^2+^ amount in the SR but decreases extracellular Ca^2+^ entry via the SOCE mechanism and cytosolic Ca^2+^ level at rest. Considering that the amount of CASQ1 in fast-twitch skeletal muscle fibers is approximately three times greater than that in slow-twitch skeletal muscle fibers [50], these influences of the CASQ1-STIM2 interaction could be much greater in fast-twitch skeletal muscle than in slow-twitch skeletal muscle fibers.

In skeletal muscle, STIM1 and Orai1 are the main SOCE-mediating proteins, and STIM2 plays fine-tuning roles in SOCE in addition to STIM1 [1,2,12,13]. The CASQ1-STIM1 interaction is mediated by the C-terminal region of CASQ1 (i.e., S1B in Figure 1B, 353–378 amino acids in mouse CASQ1, 362–387 amino acids of human CASQ1, or 352–367 amino acids of rabbit CASQ1 (known as asp-rich region) [27,35]). However, in the present study, the CASQ1-STIM2 interaction was mainly mediated by the N-terminal region of CASQ1. This difference suggests that the CASQ1-STIM2 interaction could be one of the detailed ways of STIM2 to fine-tune SOCE in skeletal muscle. On the other hand, the C-terminus of CASQ1, which covers the S1B region, also showed a significant binding ability to STIM2 (Figure 1B,D), suggesting the possibility that multiple sites of CASQ1 are involved in binding to STIM2.

Based on previous reports and the present study [23,27,35], CASQ1 is not only an internal Ca^2+^-buffering and sensing protein of the SR but also an active long-range communicator with Orai1 via binding to STIM2, although it is a trapped protein in the SR. Considering that Ca^2+^ via the SOCE mechanism plays a secondary role in skeletal muscle contraction (i.e., not for the initiation but the maintenance of the skeletal muscle contraction) [1,2,51], the long-range regulation of SOCE by the CASQ1-STIM2 interaction seems to be involved in ‘the adaptation to repetitive and/or long-term contractions of skeletal muscle’, for example, during tetanic stimulation or fatigue. Balancing interactions between CASQ1-STIM2 and Orai1-STIM2 could be an effective way to adapt to situations with the repetitive and/or long-term contractions, i.e., to save more Ca^2+^ in the SR Ca^2+^ store and to lower useful Ca^2+^ in the cytosol than in normal situations and to release Ca^2+^ from the SR to the cytosol when it is needed (Figure 5B, an adaptation model in the lower panel).

Expressing the full-length WT CASQ1 (i.e., CASQ1-STIM2 interaction) in the present study increased the Ca^2+^ amount in the SR. The increased Ca^2+^ amount in the SR has two explanations. First, it is possible that an increase in total Ca^2+^-buffering capacity by the expression of WT CASQ1 could contribute to the increased Ca^2+^ amount in the SR. However, the C1 region has a similar effect on the Ca^2+^ amount in the SR, although it almost lacks the Ca^2+^-buffering ability. Therefore, free CASQ1 may be increased in C1-expressing myotubes because, on behalf of endogenous CASQ1, the C1 region binds to STIM2, which is a similar condition to WT CASQ1 expression. Indeed, the expression of CASQ1 in C2C12 skeletal myotubes showed an increase in Ca^2+^ amount in the SR [27], and conversely, skeletal muscle fibers from the flexor digitorum brevis of CASQ1 single- or CASQ1/CASQ2 double-knock mice showed a decrease in Ca^2+^ amount in the SR [52]. Second, it is also possible that available STIM2 could be decreased by the expression of WT CASQ1 or C1 region due to additive WT CASQ1-STIM2 or C1-STIM2 interaction to endogenous CASQ1-STIM2 interaction. It was reported that STIM2 attenuates SERCA1a activity [22] (which is another different aspect of STIM2 from STIM1 because STIM2 has an opposite effect on SERCA1a activity [38]). The attenuation of SERCA1a by STIM2 could be alleviated by the decrease of available STIM2, which induces high Ca^2+^ in the SR and low Ca^2+^ in the cytosol. The less Orai1-STIM2 interaction in WT CASQ1 or C1-expressing myotubes (Figure 3D) supports the decrease of available STIM2.

STIM1 (a homolog of STIM2) or CASQ1-STIM1 interaction may be involved in the changes of Ca^2+^ movement and distribution that we present in this study. Interestingly, WT CASQ1 or C1-expressing myotubes have a similar tendency to STIM2-knockdown myotubes [22]: decreases in SOCE and cytosolic Ca^2+^ levels and increases in RyR1 activity and Ca^2+^ amount in the SR were seen. However overexpression or knockdown of STIM1 in mouse skeletal myotubes does not change the cytosolic Ca^2+^ level or Ca^2+^ amount in the SR [42], suggesting that it is highly unlikely that the changes in Ca^2+^ movement and distribution are due to STIM1 or CASQ1-STIM1 interaction. This is the other different aspect of STIM2 from STIM1.

Unlike the full-length WT CASQ1, the C1 region induced mitochondrial shape abnormalities and decreased the expression of TRPC6 and CaM1. Mild mitochondria swelling regulates mitochondrial metabolism and functions; however, the excessive mitochondria swelling seen in the present study causes mitochondrial dysfunction and is known to be induced by mitochondrial Ca^2+^ overload [53]. Mitochondria are the second intracellular Ca^2+^ store in skeletal muscle and are closely localized in triad junctions to functionally communicate with the SR [6,7,54,55]. Considering that the Ca^2+^ amount in the SR was increased by the CASQ1-STIM2 interaction and that the C1 region is almost lacking Ca^2+^-buffering ability, an excessively high Ca^2+^ amount in the SR due to the C1-STIM2 interaction could not be buffered in C1-expressing myotubes and could induce mitochondrial Ca^2+^ overload to maintain a Ca^2+^ balance between the SR and mitochondria (Figure 5B, schematic diagram on the right-hand side of the upper panel), which could be the reason for the mitochondria swelling in the C1-expressing myotubes. According to this hypothesis, the swelling phenomenon of mitochondria seems to be not fully due to the C1-STIM2 interaction but partly due to the C1 region itself. It seems that for a compensatory mechanism for the Ca^2+^ overload in the SR and mitochondria, the expression of TRPC6 (another type of SOCE-mediating channel) or CaM1 (a Ca^2+^-dependent protein) is decreased in C1-expressing myotubes (as shown in Figure 3C). Mitochondrial swelling by the C1 region is different from the swelling in another case: mitochondria with onion-shaped cristae generated by a null mutant of STIM1 (R429C, which does not mediate SOCE and causes human muscular hypotonia [41]). The study and the present study suggest the possibility that Ca^2+^ via SOCE affects mitochondrial shapes in a Ca^2+^ amount-dependent manner. The length of swelling mitochondria was decreased. This could be due to the increased expression of Drp-1, which mediates mitochondrial fission [56]. Abnormal mitochondrial shapes (and the subsequent mitochondrial dysfunctions) with abnormal SOCE have been reported in skeletal myopathies, such as congenital muscular dystrophy [23,57]. Therefore, it is possible that the CASQ1-STIM2 interaction is a linker between abnormal SOCE and mitochondrial abnormalities in skeletal myopathies and that functional interplay among CASQ1 and STIM2 and the subsequent changes in SOCE and Ca^2+^ distribution in cellular compartments are important for normal mitochondria and skeletal muscle functions. These are novel notions from this study.

Additionally, TAM is a skeletal muscle disease that is found in patients with mutated CASQ1 or CASQ1-null mice [23,30]. However, TAM phenomena were not found in the WT CASQ1- or C1-expressing myotubes, which suggests that the CASQ1-STIM1 interaction is not directly related to TAMs. CASQ1 has three almost identical domains that show topology similar to that of thioredoxin from *E. coli* (called thioredoxin-like domains) [58]. However, none of them are related to the CASQ1-STIM1 interaction because disrupting or eliminating the thioredoxin-like domains of CASQ1 had no relevance to the CASQ1-STIM2 interaction.

## 5. Conclusions

In conclusion, CASQ1 is not only a passive Ca^2+^-buffering or sensing protein within the SR but also, by interacting with STIM2, an active player in spatiotemporal cellular Ca^2+^ movements in skeletal muscle (i.e., SOCE, Ca^2+^ release for skeletal muscle contraction, and Ca^2+^ distribution). Furthermore, STIM2 in skeletal muscle is not an extra or alternative protein of STIM1 that mediates SOCE but is a functionally equivalent protein by regulating Ca^2+^-related cellular events by interacting with other proteins, such as CASQ1 rather than Orai1.

## Figures and Tables

**Table 1:** Properties of mouse primary skeletal myotubes that expressed the WT CASQ1 or the C1 region. The values, except for those of the cytosolic Ca^2+^ levels at rest, were normalized to the mean value of those from the vector control. The values are presented as the mean ± SEM for the number of myotubes shown in parentheses. * Significant difference compared with vector control (*p* < 0.05).

		Vector Control	WT CASQ1	C1
Width of myotubes		1.00 ± 0.07 (30)	0.97 ± 0.08 (30)	1.04 ± 0.10 (30)
SOCE	Peak area	1.00 ± 0.08 (50)	0.69 ± 0.11 * (50)	0.76 ± 0.10 * (50)
	Slope	1.00 ± 0.07 (30)	1.03 ± 0.09 (30)	1.02 ± 0.09 (30)
Releasable Ca^2+^ level from the SR		1.00 ± 0.08 (50)	1.45 ± 0.06 * (50)	1.24 ± 0.08 * (50)
Resting [Ca^2+^]_cytosol_, nM		94.33 ± 9.23 (50)	67.09 ± 9.15 * (50)	74.13 ± 8.48 * (50)
KCl response	Peak area	1.00 ± 0.04 (50)	1.19 ± 0.05 * (50)	1.12 ± 0.04 * (50)
	Slope	1.00 ± 0.07 (30)	1.03 ± 0.08 (30)	1.02 ± 0.10 (30)
Caffeine response	Peak area	1.00 ± 0.06 (50)	1.32 ± 0.05 * (50)	1.25 ± 0.06 * (50)
	Slope	1.00 ± 0.07 (30)	0.99 ± 0.11 (30)	0.98 ± 0.10 (30)

**Table 2:** Length of mitochondria and expression level of Drp-1 or Mfn-1 in mouse primary skeletal myotubes that expressed the WT CASQ1 or C1 region. The values are presented as the mean ± SEM for the number of mitochondria or experiments shown in parentheses. The values were normalized to the mean values of the vector control. * Significant difference compared with vector control (*p* < 0.05). ^#^ Significant difference compared with WT CASQ1 (*p* < 0.05).

	Vector Control	WT CASQ1	C1
Length of swelling mitochondria	1.00 ± 0.07 (53)	0.93 ± 0.09 (37)	0.38 ± 0.06 * ^#^ (35)
Expression level of Drp-1	1.00 ± 0.00 (3)	1.01 ± 0.05 (3)	1.39 ± 0.18 * ^#^ (3)
Expression level of Mfn-1	1.00 ± 0.00 (3)	1.00 ± 0.04 (3)	1.03 ± 0.05 (3)

## Supplementary Materials

The following are available online at https://www.mdpi.com/article/10.3390/cells10112821/s1, Table S1: PCR primers for the cloning of GST-CASQ1 or GST-CASQ1 regions (GST-A to GST-E). Table S2: PCR primers for the cloning of GST-C regions (GST-C1 to GST-C5). Table S3: PCR primers for the cloning of the C1 region into the pCMS-RFP vector. Table S4: Expression level of the proteins that mediate or regulate EC coupling, SOCE, or myotube differentiation in the WT CASQ1 or C1-expressing mouse primary skeletal myotubes.
