# Supplementary material for: Calsequestrin 1 Is an Active Partner of Stromal Interaction Molecule 2 in Skeletal Muscle
Source: Cells. 2021 Oct 20;10(11):2821. doi: 10.3390/cells10112821 (PMC8616366; doi:10.3390/cells10112821)
Supplement: Supplementary file 1 [file cells-10-02821-s001.zip › cells-1393433-supplementary.pdf]

Calsequestrin 1 is an active partner of stromal interaction molecule 2 in skeletal muscle

Seung Yeon Jeong[1,2], Mi Ri Oh[1,2], Jun Hee Choi[1,2], Jin Seok Woo[3], and Eun Hui Lee[1,2,*]

[1]Department of Physiology, College of Medicine, The Catholic University of Korea, Seoul 06591; [2]Department of Biomedicine & Health Sciences, Graduate School, The Catholic University of Korea, Seoul 06591; [3]Department of Physiology, David Geffen School of Medicine, UCLA, Los Angeles, CA 10833, USA;
*Correspondence: Eun Hui Lee (ehui@catholic.ac.kr)

List of Supplementary Materials

Supplementary Table S1. PCR primers for the cloning of GST-CASQ1 or GST-CASQ1 regions (GST-A to GST-E)

Supplementary Table S2. PCR primers for the cloning of GST-C regions (GST-C1 to GST-C5)

Supplementary Table S3. PCR primers for the cloning of the C1 region into the pCMS-RFP vector

Supplementary Table S4. Expression level of the proteins that mediate or regulate EC coupling, SOCE, or myotube differentiation in the WT CASQ1 or C1-expressing mouse primary skeletal myotubes.

Supplementary Table S1

PCR primers for the cloning of GST-CASQ1 or GST-CASQ1 regions (GST-A to GST-E)

	PCR primers (EcoR I & Xho I)	
GST-CASQ1	Forward	5' – GGAATTCATGGGGGCCAGAGCAGTG – 3'
	Backward	5' – CGCTCGAGCTAGTCGTCGTCATCATC – 3'
GST-A	Forward	5' – GGAATTCATGGGGGCCAGAGCAGTG – 3'
	Backward	5' – CGCTCGAGCTACATCTCCATCCATATG – 3'
GST-B	Forward	5' – GGAATTCGATAACGAGGAGGACCTG – 3'
	Backward	5' – CGCTCGAGCTAGTCGTCGTCATCATC – 3'
GST-C	Forward	5' – GGAATTCATGGGGGCCAGAGCAGTG – 3'
	Backward	5' – CGCTCGAGCTAAATCAACTCTACAG – 3'
GST-D	Forward	5' – GGAATTCGAAGGTGAACGAGAGC – 3'
	Backward	5' – CGCTCGAGCTACTCTAAGAACTC – 3'
GST-E	Forward	5' – GGAATTCACTCTCAAGGCTGTGG – 3'
	Backward	5' – CGCTCGAGCTAGTCGTCGTCATCATC – 3'

Supplementary Table S2

PCR primers for the cloning of GST-C regions (GST-C1 to GST-C5)

	PCR primers (EcoR I & Xho I)	
GST-C1	Forward	5’ – GGAATTCATGGGGGCCAGAGCAGTG – 3’
	Backward	5’ – CGCTCGAGCTACAGGATTAG – 3’
GST-C2	Forward	5’ – GGAATTCGAGTTAGCAG – 3’
	Backward	5’ – CGCTCGAGCTAAATCAACTCTACAG – 3’
GST-C3	Forward	5’ – GGAATTCATGGGGGCCAGAGCAGTG – 3’
	Backward	5’ – CGCTCGAGCTAATCCTTCTCTGAGTC – 3’
GST-C4	Forward	5’ – GGAATTCGCAGCTGTGGCCAAGAAA – 3’
	Backward	5’ – CGCTCGAGCTACTCGCCGTCATATTC – 3’
GST-C5	Forward	5’ – GGAATTCTTTTCTGCAGAC – 3’
	Backward	5’ – CGCTCGAGCTAAATCAACTCTACAG – 3’

Supplementary Table S3

PCR primers for the cloning of the C1 region into the pCMS-RFP vector

	PCR primers (EcoR I & Sal I)	
C1 region	Forward	5’- GGAATTCATGGGGGCCAGAGCAGTG -3’
	Backward	5’- GCGTCGACTCACAGGATTAGCTCC -3’

Supplementary Table S4

Expression level of the proteins that mediate or regulate EC coupling, SOCE, or myotube differentiation in the WT CASQ1 or C1-expressing mouse primary skeletal myotubes. α-actin was used as a loading control. Three independent experiments per protein were conducted. The values are presented as the mean ± SEM for three independent experiments. The expression level of protein was normalized to the mean value of the vector control. *Significant difference compared with vector control ($p < 0.05$). #Significant difference compared with WT CASQ1 ($p < 0.05$).

	Vector control	WT CASQ1	C1
MyoD	1.00 ± 0.00	1.02 ± 0.09	0.98 ± 0.06
Myogenin	1.00 ± 0.00	0.97 ± 0.07	1.00 ± 0.09
RyR1	1.00 ± 0.00	1.01 ± 0.08	0.98 ± 0.08
DHPR	1.00 ± 0.00	1.01 ± 0.13	1.02 ± 0.13
SERCA1a	1.00 ± 0.00	0.97 ± 0.15	0.97 ± 0.13
TRPC1	1.00 ± 0.00	0.98 ± 0.13	1.02 ± 0.12
TRPC3	1.00 ± 0.00	1.01 ± 0.08	0.98 ± 0.08
TRPC4	1.00 ± 0.00	1.00 ± 0.15	0.99 ± 0.13
TRPC6	1.00 ± 0.00	1.02 ± 0.09	0.71 ± 0.11 *, #
TRIM32	1.00 ± 0.00	0.97 ± 0.09	0.98 ± 0.08
Orai1	1.00 ± 0.00	1.02 ± 0.07	1.03 ± 0.04
STIM1	1.00 ± 0.00	0.98 ± 0.07	0.99 ± 0.11
STIM2	1.00 ± 0.00	1.02 ± 0.07	1.04 ± 0.09
CSQ1	1.00 ± 0.00	1.00 ± 0.07	1.01 ± 0.07
JP1	1.00 ± 0.00	0.99 ± 0.09	1.01 ± 0.07
JP2	1.00 ± 0.00	1.04 ± 0.11	0.97 ± 0.09
CaM1	1.00 ± 0.00	0.98 ± 0.08	0.61 ± 0.17 *, #
